# A case report of necrotizing enterocolitis in a moderately preterm neonate with LCHADD—A call to focus on the basics while utilizing advanced new therapies

**DOI:** 10.3389/fped.2023.1081802

**Published:** 2023-02-13

**Authors:** Marina Metzler, William Burns, Carly Mitchell, Stephanie Napolitano, Bimal P. Chaudhari

**Affiliations:** ^1^ Pediatric Residency, Nationwide Children’s Hospital, Columbus, OH, United States; ^2^ Division of Genetic and Genomic Medicine, Nationwide Children’s Hospital, Columbus, OH, United States; ^3^ Division of Neonatology, Nationwide Children’s Hospital, Columbus, OH, United States; ^4^ Department of Pediatrics, College of Medicine, Ohio State University, Columbus, OH, United States; ^5^ Steve and Cindy Rasmussen Institute for Genomic Medicine, Nationwide Children’s Hospital, Columbus, OH, United States

**Keywords:** necrotizing enterocolitis, triheptanoin, preterm, human milk, LCHADD

## Abstract

Long-chain 3-hydroxyacyl-CoA dehydrogenase deficiency (LCHADD) is an autosomal recessive condition of impaired beta-oxidation. Traditionally, treatment included restriction of dietary long-chain fatty acids *via* a low-fat diet and supplementation of medium chain triglycerides. In 2020, triheptanoin received FDA approval as an alternative source of medium chain fatty acids for individuals with long-chain fatty acid oxidation disorders (LC-FAOD). We present a case of a moderately preterm neonate born at 33 2/7 weeks gestational age with LCHADD who received triheptanoin and developed necrotizing enterocolitis (NEC). Prematurity is known as a major risk factor for NEC, with risk increasing with decreasing gestational age. To our knowledge, NEC has not previously been reported in patients with LCHADD or with triheptanoin use. While metabolic formula is part of the standard of care for LC-FAOD in early life, preterm neonates may benefit from more aggressive attempts to use skimmed human milk to minimize exposure to formula during the risk period for NEC during feed advancement. This risk period may be longer in neonates with LC-FAOD compared to otherwise healthy premature neonates.

## Introduction

Long-chain fatty acid oxidation disorders (LC-FAOD) are rare, autosomal recessive, conditions of impaired beta-oxidation (1–4). Isolated long-chain 3-hydroxyacyl-CoA dehydrogenase deficiency (LCHADD) is the most common mitochondrial trifunctional protein (MTP) complex deficiency with an estimated incidence of 1:363,768 for LCHADD and 1:1,822,568 for MTP deficiency in the United States (1, 5, 6). The c.1528G > C variant in *HADHA* is the most frequently associated allele in LCHADD with 87%–90% of symptomatic European-ancestry individuals carrying at least one copy (1, 7). Its presentation can include hypoketotic hypoglycemia, lactic acidosis, liver dysfunction, encephalopathy, rhabdomyolysis, fatigue, and cardiac arrhythmias (1, 2, 4, 5, 8–10). It is also associated with prematurity and maternal hemolysis, elevated liver enzymes, and low platelets (HELLP) syndrome (1, 11). LCHADD is part of the newborn screen in many North American and European countries, including the United States (1, 5, 8). Treatment includes restriction of dietary long-chain fatty acids, supplementation with medium chain triglycerides (MCT), and low-fat diet (1, 2, 3, 4, 8). In 2020, triheptanoin, a synthetic MCT, received FDA approval as an alternative source of medium chain fatty acids for children and adults with LC-FAOD (2). Triheptanoin directly enters mitochondria for beta-oxidation with resulting metabolites providing energy as well as replenishing key Krebs cycle intermediates and has proven to be more effective than MCT oil in reducing risk of cardiomyopathy and rhabdomyolysis (3, 10, 12).

Necrotizing enterocolitis (NEC) is a known complication of prematurity and significant cause of morbidity and mortality (13, 14). We present a case of NEC in a 9-day-old, moderately preterm male with confirmed LCHADD who received triheptanoin. Triheptanoin treatment is described in neonates as young as 2 and 3 days old (15, 16). However, the youngest age of reported use in an infant born prematurely is 13 months (11). While NEC has been reported as the presentation of MTP deficiency in a 3-day-old female born at 35 weeks, to our knowledge, there have been no published, peer-reviewed reports of NEC developing in patients with LCHADD or after the initiation of triheptanoin (5).

## Case description

**Figure 1:**
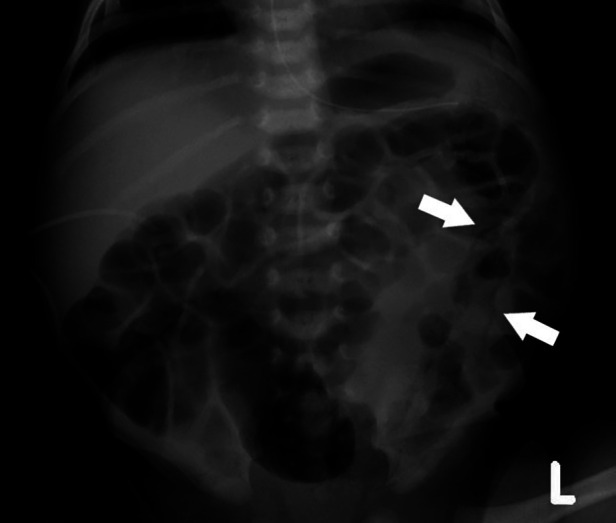
Abdominal x-ray demonstrating pneumatosis intestinalis.

**Figure 2:**
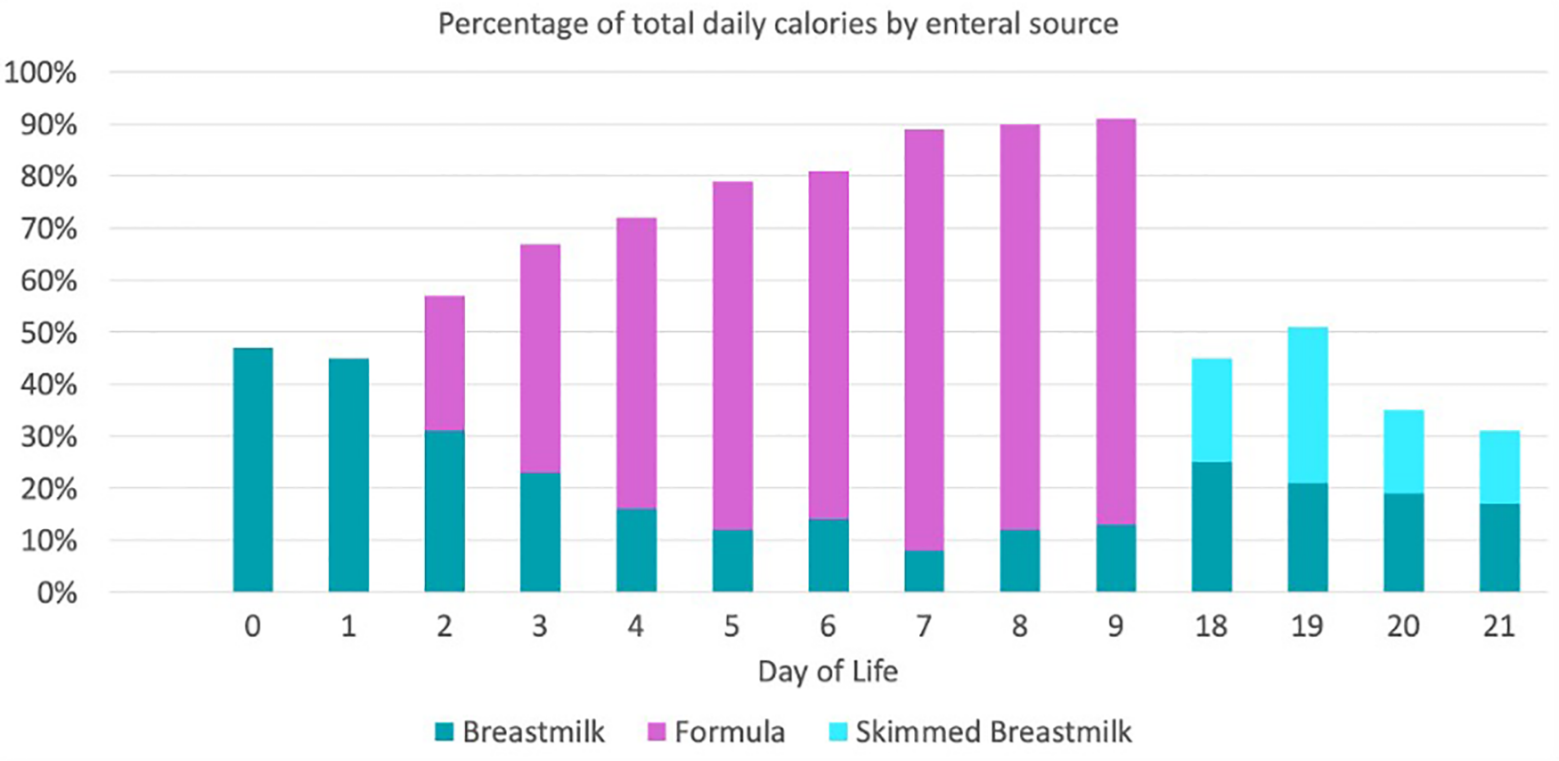
Enteral feeding advancement regimen prior to developing NEC. *SolCarb was added starting on day of life 5. *Similac liquid protein fortifier was added starting on day of life 7.

This baby boy was born at 33 2/7 weeks gestational age to a 36-year-old gravida 3, para 3 mother with negative serologies, except for Group B Streptococcus unknown, whose pregnancy was complicated by medication-controlled gestational diabetes and gestational hypertension, and a family history significant for a sibling with LCHADD. The patient was delivered by cesarean section at an outside facility due to concern for serious maternal morbidity associated with preeclampsia, specifically HELLP syndrome. Neonatal resuscitation was unremarkable and the Appearance, Pulse, Grimace, Activity, and Respiration (APGAR)s were 8 and 9 at 1 and 5 min, respectively. The birth weight was 2.46 kg, length 47.5 cm, and head circumference 32.5 cm, which were all appropriate for gestational age. The patient was admitted to the level III NICU at the birth facility. A peripheral intravenous line was placed and 10% dextrose fluids at 150 ml/kg/day were initiated. At six hours of life, based on the significant family history and the maternal HELLP syndrome frequently seen in mothers with gestations complicated by LCHADD, our patient was transported to our level IV NICU for metabolic consultation and further evaluation and management. On admission, serum acylcarnitine profile was consistent with LCHADD given elevation of C16-OH and C18-OH levels with the ultimate diagnosis based on perinatal and family history in conjunction with biochemical and molecular studies (Table 1). Echocardiogram shortly after arrival showed a small patent ductus arteriosus and patent foramen ovale. Enteral nutrition with donor human milk was initiated at 12 h of life. Triheptanoin was initiated at 14 h of life at a dose of 0.5 g/kg/day and advanced 0.5 g/kg every 5–7 days. As feeds were advanced by unit standard of 20 ml/kg/day, he was limited to 23 ml/kg/day of human milk with the remainder of his enteral diet consisting of Enfaport (Chicago, IL, United States), SolCarb (Westbury, NY, United States), and Similac liquid protein fortifier (Abbott Park, IL, United States) formulas (Table 2). His feeds were fortified to 24 kcal/oz on day of life 6. On day 7 of life, the patient started to have new bradycardia and desaturation episodes with two small emeses but a reassuring abdominal examination. Enteral nutrition was continued. On day 9 of life, he had one episode of emesis and then hematochezia. An abdominal x-ray (Figure 1) demonstrated pneumatosis intestinalis and he was subsequently diagnosed with NEC (modified Bell Stage IIa). He was managed conservatively with bowel rest, ampicillin–sulbactam and gentamicin for 7 days, and bowel decompression for 5 days. He was maintained on total parental nutrition (TPN) with 4 g/kg/day TrophAmine, glucose infusion rate ∼15 mg/kg/min, and 2 g/kg/day 20% Intralipid. Diet following re-initiation of enteral nutrition consisted of a combination of skimmed and full fat maternal and donor human milk, Enfaport, MCT oil instead of triheptanoin (per parental request), SolCarb, and Similac liquid protein fortifier (Figure 2). He recovered uneventfully and was discharged home on day 33 of life.

**Table 1:** Initial biochemical and molecular lab results.

Initial Labs	Ammonia: 90 µmol/L Glucose: <20 mg/dl	Liver transaminases: normal Creatinine kinase: normal
Acylcarnitine profile	Newborn screen	C16-OH: 0.610 µmol/L
	Serum	C16-OH: 0.61 µmol/L C18: 1.35 µmol/L C18-OH: 0.74 µmol/L C18_1-OH: 0.30 µmol/L
Sanger sequencing of *HADHA*	Variants	Interpretation	Inheritance	ACMG criteria
	c.2225_2228dup (p.Phe744Thrfs*10)	Pathogenic	Parental studies not performed. Phase determined on sibling testing.	PM3, PP4, PM2
	c.1528G > C (p.Glu510Gln)	Pathogenic		PM3, PP1, PP3, PP4

**Table 2:** Enteral feeding advancement regimen prior to developing NEC.

Day of life	Total calories per day (kcal)	Triheptanoin intake (g/kg/day)	Triheptanoin intake (%) of total calories	MCT intake (%) of total calories	Protein (g)		Fat (g)	Carbohydrate (g)	% calories from breastmilk	% calories from formula
					Parenteral	Enteral				
0	75	0.48	13%	15%	4.5	0.5	3.3	3.6	47%	0%
1	119	0.48	8%	10%	5	0.8	4.5	15.7	45%	0%
2	148	0.48	7%	17%	5	2	6.1	18.1	31%	26%
3	185	0.48	5%	23%	5	3.5	8.2	21.6	23%	44%
4	217	0.48	6%	26%	5	4.7	9.9	24.8	16%	56%
5^a^	243	0.96	8%	29%	5	5	11.3	27.4	12%	67%
6	273	1.12	9%	26%	5	5	12	33	14%	67%
7^b^	280	1.12	8%	26%	0	8.8	10.8	28.9	8%	81%
8	338	1.12	7%	23%	0	10	12.8	47.9	12%	78%
9	353	1.6	10%	25%	0	10	14.6	47.2	13%	78%

NEC, necrotizing enterocolitis; MCT, medium chain triglycerides.

^a^ SolCarb was added starting on day of life 5.

^b^ Similac liquid protein fortifier was added starting on day of life 7.

## Discussion

NEC is a spectrum of gastrointestinal disease with incompletely understood pathophysiology (17, 18). NEC occurs in a gut compromised by intestinal immaturity with an inciting mucosal injury, disruption to the microvascular supply, alteration in the microbial colonization, activation of inflammatory processes, and genetic predisposition (14, 17, 19). NEC occurs in 1%–5% of all NICU admissions and 5%–10% of all very low birthweight infants (<1,500 g) (20). Validated NEC risk factors include being small for gestational age, low gestational age, sepsis, assisted ventilation, premature rupture of membranes, black race, out born status, and delayed gut priming or initiation of feeds (17, 21, 22). However, there are several pre- and post-natal NEC risk factors beyond this list specific to our case. Data for a link between maternal preeclampsia, as seen with HELLP syndrome, and NEC have been mixed with some studies showing an increased NEC risk while other studies attributing this trend to delivery at an earlier gestational age for maternal wellbeing (19, 23). Research has shown long-chain 3-hydroxyacyl-CoA dehydrogenase to be expressed during fetal development in fetal heart, eye, liver, brain, and gut epithelium (9). It has also been suggested that fatty acid oxidation enzymes in the gut play a role in mucus synthesis and intracellular junction integrity (5). These findings indicate that gut development could already be impaired in neonates with LCHADD, leading them to be more susceptible to NEC. Given that LCHADD is rare and neonates with LCHADD are typically born at gestational ages when NEC is infrequent, it may be challenging, if not impossible, to quantify this risk using traditional epidemiologic methods.

Postnatally, NEC prevention has focused on human milk feeding. The strong support for early human milk feedings arises from bioactive substances such as lactoferrin and human milk oligosaccharides with various bactericidal, immunomodulating, and intestinal maturation-inducing properties found in human milk (13, 17, 20, 24–26). NEC rates were found to be six times more common in premature infants exclusively formula fed compared to those fed exclusively human milk and 3-times more common in those fed formula exclusively compared to those fed a mixture of formula and human milk (26, 27). There has been an increasing amount of literature looking specifically at the role fatty acids in human milk play in gut development, microbial colonization, immune function, and inflammatory response in *in vitro*, *in vivo*, and in human cohort studies (28–30). One recent example found less NEC among a cohort of preterm neonates that received supplementation of the specific long-chain polyunsaturated fatty acid (LCPUFA) docosahexaenoic acid (DHA) compared to controls (31). While this supplementation is exciting for future work, current expert-consensus NEC prevention targets include supporting antenatal corticosteroids before delivery, prioritizing mother's own milk, a standardized unit-based feeding protocol, use of programmatic approach to reducing NEC with quality improvement methodology, discussing risks and benefits of probiotic administration with parents, and skin-to-skin care (24). Modest evidence supports other standards of care with donor human milk as a substitute for mother's own milk over formula, limiting prolonged empiric antibiotic courses, and limiting the use of histamine-2 antagonists (22, 24). As our knowledge of NEC pathophysiology grows, it is natural to target preventative and treatment strategies to the patient's specific circumstances and disease (32).

In almost all cases, preference for a human milk-based diet seems prudent. We used formula early in this patient's course due to his metabolic needs specific to his LC-FAOD. However, upon reintroduction of the feeds after NEC treatment, we utilized skimmed milk to maximize its NEC protection benefits while minimizing its metabolic risk to our patient. Skimmed human milk to eliminate long-chain triglycerides has been used in the treatment of infants with chylothorax (33). Milk skimming techniques include gravity, centrifuge, top loading washing machine acting as a centrifuge, and a commercially available cream separator (16, 33, 34). The effectiveness of milk skimming has been compared between centrifuged refrigerated milk, centrifuged non-refrigerated milk, and non-centrifuged refrigerated milk stored for 24 h. The centrifuged refrigerated method was most effective at skimming while retaining protein and immunoglobulins (33). While centrifuge-based methods are most effective, non-centrifuge methods do not require additional equipment and may be more logistically feasible for families. However, our patient's family needed to cautiously transport the refrigerated mother's milk to the hospital while maintaining gravity separation and staff needed to budget time for skimming to the already short shelf life of fresh human milk. In the end, our patient received primarily donor milk skimmed by centrifuge and a minority of gravity skimmed mother's milk during the initial hospitalization due to these logistical challenges.

Skimmed term human milk contains 12.4 kcal/oz and 0.4 g/dl fat compared to unskimmed term human milk that contains 20 kcal/oz and 3.6 g/dl. Skimmed milk would, therefore, require fortification to increase calories and any nutritional deficiencies not provided in the skimmed milk. The specific metabolic needs leading to formula use need to be balanced with the needs of a premature infant. Earlier adoption of a diet maximizing human milk usage and minimizing formula may have helped prevent NEC with the protective factors of human milk while meeting the specific dietary restrictions of our patient. Based on the research supporting LCPUFA, this skimming may decrease some of the NEC-protective benefits of human milk. However, we believe the remaining properties of human milk likely make it a superior choice to formula in this unusual clinical setting. Further research would be needed to assess this theory.

A review of the literature failed to identify other reports of NEC after early neonatal use of triheptanoin. Our decision to begin standard MCT oil following the episode of NEC was driven by parental preference and paucity of data on triheptanoin in this setting. From a medical standpoint, MCT has been used extensively without known complications of NEC although it is less effective in reducing adverse events of LC-FAODs compared to triheptanoin (4, 35). With the parental concerns regarding triheptanoin and our patient's complication, the decision was made to switch to MCT with its better described safety profile despite known differences in efficacy. Given the challenge of studying an uncommon complication (NEC) of a rare group of disorders (LC-FAODs) and the relatively limited data on triheptanoin (FDA approved in 2020), case reports such as this are likely to comprise a significant portion of the evidence basis for assessing a possible link between triheptanoin and NEC. Making that assessment also requires consideration of alternative causes of NEC in our patient and potential opportunities for NEC prevention in similarly situated neonates.

In summary, we present a case of a moderately preterm neonate with LCHADD who developed NEC. We do not think the triheptanoin was the cause of NEC. Limited use of human milk in the setting of metabolic management for LC-FAOD may have contributed to the development of NEC. It is important to consider skimmed human milk to minimize exposure to formula in the NEC risk period during feeding advancement, which may be longer in neonates with LC-FAOD than in otherwise healthy premature neonates.

## Data Availability

The original contributions presented in the study are included in the article/Supplementary Material, further inquiries can be directed to the corresponding author.
